# The UK National Recovery Survey: nationally representative survey of people overcoming a drug or alcohol problem

**DOI:** 10.1192/bjo.2023.654

**Published:** 2024-03-14

**Authors:** Ed Day, Ifigeneia Manitsa, Amanda Farley, John F. Kelly

**Affiliations:** School of Psychology, Institute for Mental Health, University of Birmingham, UK; Institute for Applied Health Research, University of Birmingham, UK; Harvard Medical School and Center for Addiction Medicine, Recovery Research Institute, at Massachusetts General Hospital, Boston, USA

**Keywords:** Alcohol use disorder, drug use disorder, recovery, problem resolution, treatment

## Abstract

**Background:**

Alcohol or drug (AOD) problems are a significant health burden in the UK population, and understanding pathways to remission is important.

**Aims:**

To determine the UK population prevalence of overcoming an AOD problem and the prevalence and correlates of ‘assisted’ pathways to problem resolution.

**Method:**

Stage 1: a screening question was administered in a national telephone survey to provide (a) an estimate of the UK prevalence of AOD problem resolution; and (b) a demographic profile of those reporting problem resolution. Stage 2: social surveying organisation YouGov used the demographic data from stage 1 to guide the administration of the UK National Recovery Survey to a representative subsample from its online panel.

**Results:**

In stage 1 (*n* = 2061), 102 (5%) reported lifetime AOD problem resolution. In the weighted sample (*n* = 1373) who completed the survey in stage 2, 49.9% reported ‘assisted’ pathway use via formal treatment (35.0%), mutual help (29.7%) and/or recovery support services (22.6%). Use of an assisted pathway was strongly correlated with lifetime AOD diagnosis (adjusted odds ratio [AOR] = 9.54) and arrest in the past year (AOR = 7.88) and inversely correlated with absence of lifetime psychiatric diagnosis (AOR = 0.17). Those with cocaine (AOR = 2.44) or opioid problems (AOR = 3.21) were more likely to use assisted pathways compared with those with primary alcohol problems.

**Conclusion:**

Nearly three million people have resolved an AOD problem in the UK. Findings challenge the therapeutic pessimism sometimes associated with these problems and suggest a need to learn from community-based self-change that can supplement and enhance existing treatment modalities.

The construct of ‘recovery’ from problematic alcohol or drug (AOD) use is beginning to gain prominence and is generally thought to involve two components:^1^ (a) remission from problematic AOD use, meaning either abstinence or controlled use without symptoms; and (b) good mental and physical health alongside involvement in the ‘rights, roles and responsibilities of society’.^2^ Although the term ‘recovery’ has been an aspiration of government policy around both alcohol^3^ and drug use^4^ problems for more than a decade, the prevalence of people who have overcome an AOD problem has never been estimated in the UK population. A recent study in the USA reported that 9% of a general population sample had previously had a problem with AOD but no longer did,^5^ but it is likely that the situation in the UK is different for a variety of social, political and cultural reasons.^6^ Pathways to recovery may involve accessing professional treatment services and medication, mutual-help groups such as Alcoholics Anonymous or Narcotics Anonymous, peer-led recovery support services, or self-change without any formal or informal support.^7^ In high-income countries in Europe and North America, fewer than 10% of those meeting criteria for alcohol or other drug use disorders receive formal treatment in any given year,^8^ and the term ‘natural recovery’ is often used to describe recovery from these disorders without professional^9^ or any other form of structured help (including participation in a mutual-help group).^5^ Despite some work on natural recovery^10,11^ and recovery in general,^7^ there has been relatively little research on either in populations outside North America. It has been well established that ceasing tobacco smoking without treatment is common,^12^ and self-change from alcohol use disorders also appears to be more common than treatment-assisted recovery.^13^ However, research exploring untreated remission from AOD use is relatively rare^14,15^ and has tended to use convenience samples of former AOD users that do not allow estimation of the prevalence of recovery.

## Aims

This study (the UK National Recovery Survey) follows the work of North American researchers in taking a population-level, public health perspective in exploring how individuals resolve a range of AOD problems.^5^ Our survey aimed to capture a nationally representative sample of people from the UK population who reported overcoming an AOD problem. The survey assessed the types of psychoactive substances that respondents used, the methods and resources deployed to overcome their problem, and whether they saw themselves as being ‘in recovery’.^16^ This paper describes the study design, gives the prevalence of self-reported AOD problem resolution, and compares lifetime use of ‘assisted’ (i.e. formal treatment/medications, recovery support services/mutual-help organisations) versus ‘unassisted’ resolution pathways. Three research questions are posed: (a) what is the prevalence of AOD problem resolution in the UK population; (b) what is the prevalence of assisted versus unassisted AOD problem resolution; and (c) what are the predictors of using assisted versus unassisted pathways?

## Method

### Sampling and data collection methods

#### Eligibility

The UK National Recovery Survey was modelled on a similar process conducted in the USA in 2017.^5^ The target population was the general population in the UK (England, Scotland, Wales and Northern Ireland) aged 18 or over who answered ‘yes’ to the screening question ‘Did you use to have a problem with drugs or alcohol, but no longer do?’. The survey was conducted by the market research and data analytics company YouGov, and ethical approval was obtained from the University of Birmingham Science, Technology, Engineering and Mathematics Ethical Review Committee (ERN_21_0565).

#### Recruitment

In stage 1, the screening question was administered in a UK nationally representative telephone omnibus survey in December 2021. The question was run twice to generate 2000 responses. This provided (a) an estimate of the prevalence of AOD problem resolution and (b) the demographic profile (such as age, gender, social grade, region) of those who reported problem resolution. These data were used to create representative sample frames of the UK population who have resolved a problem with AOD, which were then used to sample and weight the data in stage 2.

Stage 2 involved the administration of the screening question on the YouGov online panel of 400 000 active panellists in the UK in January 2022, allowing targeting of the survey to those who qualified. An active sampling method was used to draw a subsample from this panel that was representative of the group under study in terms of the sociodemographic factors elicited in stage 1. YouGov has a proprietary, automated sampling system that invites respondents based on their profile information and alignment with targets for surveys that are currently active. Respondents were automatically randomly selected based on survey availability and how that matched their profile information. Respondents were contacted by email and invited to take part in an online survey without knowing the subject at that stage. A brief, generic email invitation was used, which informed the respondent only that they were invited to participate in a survey. This helped to minimise bias from those opting in/out based on level of interest in the survey topic. The full survey was then administered online. All participants gave informed consent via the YouGov webpage prior to completing the survey.

#### Weighting

Weighting was used to adjust the contribution of individual respondents to the aggregated data, making the online survey population more representative of the national population who had overcome an AOD problem by forcing it to mimic the distribution of that larger population's significant characteristics. The stage 2 sample was weighted to be representative of all UK adults who had overcome an AOD problem by age, gender, region and social grade, based on the initial nationally representative telephone survey in stage 1. The weighting was applied to clean data at the end of the data processing phase. YouGov used random iterative method weighting as its standard approach, as there were several different standard weights that all had to be applied together. This method calculated weights for each individual respondent from the targets and achieved sample sizes for all the quota variables. The weights were recalculated several times in an iterative process until the required degree of accuracy was reached.

### Measures

#### Demographics

Sex, age, ethnicity, employment status, academic qualifications, annual income, and living accommodation and arrangements were all captured as part of the YouGov panel process.

#### Problem resolution pathway (assisted versus unassisted)

Participants were categorised as having followed an ‘assisted’ resolution pathway if they reported lifetime use of any of the following professional or peer-led services: (a) professionally led substance use disorder treatment (e.g. from a primary care physician, out-patient or in-patient/residential service); opioid agonist treatment (e.g. methadone or buprenorphine); relapse prevention/craving medication (e.g. acamprosate or naltrexone); mutual-help groups (e.g. Alcoholics Anonymous, Narcotics Anonymous, SMART Recovery); and other community-based recovery support where trained staff typically aid in service provision (e.g. sober living environments, faith-based recovery services or recovery community centres). This decision to classify the less formal services (e.g. mutual-help group participation) in the ‘assisted’ pathway followed the analysis conducted by Kelly et al,^5^ given that such participation involves engagement with a structured group and one-to-one process with a clearly delineated recovery programme and specific prescribed practices. Participants were categorised as having followed an ‘unassisted’ resolution pathway if they reported never having used any of these services; this group might be thought of as having achieved ‘natural recovery’.

#### AOD and recovery-related characteristics

Items from the Form-90^17^ were used to determine (a) whether participants considered each reported substance to be a problem, (b) age of first use (which was dichotomised as <15 *v.* ≥15 years) and (c) primary substance.^18^ Participants were also asked how long it had been since they had resolved their problem (split into three groups: 0–5 years; 5–15 years; 15+ years). The survey included items about history of 18 psychiatric disorders, including alcohol use disorder and other drug use disorder (‘Which of the following substance use and/or mental health conditions have you ever been diagnosed with?’). Criminal justice history was assessed with an item adapted from the Form-90,^17^ ‘Have you ever been arrested?’. Possible responses included ‘no’, ‘yes – in the past year’ and ‘yes – but not in the last year’.

### Statistical analysis

We calculated weighted frequencies and cross-tabulations to describe the sample, use of treatment and support services, and relationships between individual characteristics (both demographic and clinical) and resolution pathways (‘assisted’ versus ‘non-assisted’). We then performed univariate logistic regression to identify specific individual factors associated with choice of an assisted recovery pathway. Finally, we conducted multivariable analyses, where adjusted odds ratios were used to describe the relationship of the predictor of interest, adjusting for gender, age and ethnicity. All analyses were conducted using SPSS version 29.

### Ethics statement

The authors assert that all procedures contributing to this work comply with the ethical standards of the relevant national and institutional committees on human experimentation and with the Helsinki Declaration of 1975, as revised in 2008. All procedures involving human subjects/patients were approved by the University of Birmingham Technology, Engineering and Mathematics Ethical Review Committee (ERN_21_0565).

## Results

### Overall prevalence of resolved AOD problems and associated demographics and clinically relevant characteristics

Of those in the telephone survey from stage 1 (*n* = 2061), 102 (5%) individuals reported resolving an AOD problem in their lifetime (61 (3%) alcohol, 50 (2%) illicit drugs, 24 (1%) prescription drugs). The data reported here are from stage 2 of the process, which produced a sample of 1373 individuals from the YouGov online panel who completed the UK National Recovery Survey questionnaire. As described in the Method section, this sample was weighted to reflect the demographics of the sample from stage 1. As shown in Table 1, respondents who had resolved an AOD problem tended to be male, aged 25–49 years, White, employed (full-time or part-time) and living with family or relatives (the majority were living with a spouse or partner). At the time of the survey, 90% of respondents resided in England, 5% in Scotland, 4% in Wales and 1% in Northern Ireland. Most respondents had a household income which was less than £20 000 per year (30.2%) or £20–39 000 per year (29.9%).

**Table 1:** Characteristics of UK adults who endorsed ‘used to have a problem with alcohol or drugs, but no longer do’

Demographics	Weighted %	s.e.
Sex		
Male	65.0	1.46
Female	35.0	1.46
Age, years		
18–24	10.2	1.10
25–49	62.1	1.51
50–64	18.7	1.09
65+	9.0	0.78
Ethnicity		
White	90.4	0.96
Non-White	9.6	0.96
Academic qualifications		
None	5.5	0.67
Below degree level	45.6	1.63
Above degree level	45.1	1.62
Missing	3.8	0.72
Household earnings per year		
<£20k	30.2	1.48
£20k−39k	29.9	1.47
£40k−59k	15.8	1.15
£60k+	12.3	1.05
Missing	11.7	1.07
Employment status		
Working full- or part-time	62.1	1.54
Student	3.2	0.57
Retired	10.3	0.84
Unemployed	9.9	1.00
Not working	8.6	0.88
Missing	6.0	0.75
Living arrangement		
Living with a spouse or partner	37.7	1.54
Living with friend(s) or housemate(s)	6.3	0.79
Living with parent(s) or adult family member(s)	14.0	1.17
Not living with any other adult(s)	21.8	1.30
None of these	2.9	0.53
Missing	17.3	1.28
Living accommodation		
Own home outright	13.2	0.94
Own home with a mortgage	20.7	1.25
Part-own home through shared scheme	2.3	0.48
Rent from private landlord	26.8	1.47
Rent from local authority	10.2	1.00
Rent from housing association	12.7	1.08
Live with parents/family/friends but pay some rent	7.9	0.94
Live with parents/family/friends and pay no rent	4.5	0.69
Other	1.7	0.52
Accommodation location
Urban	81.6	1.19
Town and fringe	9.1	0.92
Rural	8.3	0.81
Unknown	1.0	0.26

Clinical variables	Weighted %	s.e.
Time since problem resolution		
0–5 years	53.6	1.60
5–15 years	23.6	1.38
15+ years	11.9	0.88
Did not indicate	10.9	1.00
Age at onset of first substance use		
<15 years of age	54.8	1.59
≥15 years of age	45.2	1.59
Age at onset of primary problem substance		
<15 years of age	41.3	1.60
≥15 years of age	58.7	1.60
Number of substances ever identified as a problem		
1 substance	64.5	1.58
2 substances	19.7	1.33
3+ substances	15.9	1.21
Primary problem substance		
Alcohol	57.6	1.60
Cannabis	19.8	1.34
Cocaine	5.9	0.77
Opiates	6.7	0.78
Amphetamine	4.2	0.64
Other substances	5.7	0.80
Lifetime mental health diagnoses		
Alcohol or substance use disorder	22.6	1.36
Mood disorder	23.4	1.37
Anxiety disorder	36.5	1.55
Post-traumatic stress disorder	11.8	1.03
Other mental health disorder	23.2	1.33
Never been diagnosed with a mental health disorder	25.6	1.41
Not sure	6.8	0.83
Prefer not to say	3.6	0.65
Ever been arrested		
In the past year	3.4	0.59
Yes, but not in past year	36.7	1.55
No	58.1	1.59
Prefer not to say	1.8	0.40

The most common primary problem substance was alcohol (57.6%), followed by cannabis (19.8%). Approximately half of the respondents had characteristics suggestive of more severe AOD problems, such as use of alcohol or drugs before the age of 15 (54.8%) and use of more than three substances 10+ times in their lifetime (48.3%). Just over four in ten respondents (41.3%) had started using their primary problem substance before the age of 15. Almost two-thirds (64%) had also been diagnosed with a mental health condition at some point in their life, with anxiety disorder being the most prevalent (36.5%), and 40.1% had been arrested at some point in the past. Slightly over half (53.6%) had resolved their AOD problem within the past 0–5 years, 23.6% within the past 6–15 years and 10.9% more than 15 years ago.

### Prevalence of assisted versus unassisted problem resolution pathways and prevalence of use of treatment and recovery support services

As shown in Table 2, approximately half of the respondents (49.9%) reported ever receiving assistance to help resolve their problem with AOD: 17.7% had attended their general practitioner surgery, 25.6% had received specialist treatment (out-patient or in-patient), 22.6% had accessed recovery support services and 29.7% had ever attended some form of mutual-help meeting. Combinations of these various forms of help were most common, with 19.9% receiving both specialist treatment and attending mutual-help groups. More than 20% of respondents used non-mutual-aid recovery support services, with Lived Experience Recovery Organisations^19^ being the most used type of support group (15.3%). In addition, 15.1% of the respondents reported using anti-relapse/craving medication, with 11.8% using alcohol relapse prevention treatment and 7.8% using opioid agonist treatment.

**Table 2:** Recovery pathway choices of UK adults who ‘used to have a problem with alcohol or drugs, but no longer do’

Pathway	Weighted %	s.e.
Used support	**49.9**	1.16
Formal treatment (any)	**35.0**	1.52
Specialist community addiction treatment	13.8	1.03
General practitioner	17.7	1.24
In-patient	6.8	0.78
Residential rehabilitation	5.0	0.65
Anti-relapse/craving medication	**15.1**	1.15
Alcohol	**11.8**	1.05
Acamprosate	3.0	0.55
Naltrexone	2.9	0.56
Nalmefene	2.4	0.53
Topiramate	1.5	0.40
Disulfiram	2.4	0.45
Baclofen	0.7	0.27
Opioid	**7.8**	0.86
Methadone	3.5	0.56
Buprenorphine	4.3	0.66
Suboxone (BP-naloxone)	2.2	0.49
Naltrexone	1.4	0.34
Recovery support services	**22.6**	1.35
Sober living house	5.3	0.79
Recovery school/university recovery programme	3.4	0.59
Faith-based	5.5	0.75
Lived Experience Recovery Organisation	15.3	1.15
Mutual-help groups	**29.7**	1.46
Alcoholics Anonymous	18.3	1.20
Narcotics Anonymous	8.2	0.87
Cocaine Anonymous	6.4	0.84
Gamblers Anonymous	4.2	0.73
SMART Recovery	6.9	0.86

### Correlates of assisted AOD problem resolution

Use of one or more ‘assisted’ pathways was significantly higher among ethnic minorities; in participants who first used substances at less than 15 years of age; when opiates, cocaine or other substances (benzodiazepines, hallucinogens and new psychoactive substances) were the primary problem substance; when the participant had been diagnosed with a mental health disorder at some point in their life (alcohol or substance use disorder, mood disorder, anxiety disorder and post-traumatic stress disorder); and if the participant had ever been arrested. Use of ‘assisted’ pathways was significantly lower in participants who had never received a mental health diagnosis.

All of these effects also held after adjustment for age, gender and ethnicity in multivariable models (Table 3). By far the strongest correlates of choosing an assisted pathway, as indicated by the models’ semi-partial *R*^2^ values, were lifetime diagnosis of a substance use disorder and history of arrest (either in the past year or ever). Not receiving a lifetime diagnosis of a mental health disorder was strongly associated with not using an assisted pathway.

**Table 3:** Factors associated with choosing assisted (49.9%) versus unassisted problem resolution

Variable	Assisted		Univariable			Multivariable (controlling for age, gender and ethnicity)		
	%	s.e.	OR^a^	95% CI	*R*^2^	AOR^a^	95% CI	*R*^2^
Demographics								
Gender					0.00			0.02
Male	51.2	2.11	1.00	[Reference]		1.00	[Reference]	
Female	47.5	2.42	0.86	(0.69, 1.08)		0.91	(0.72, 1.16)	
Age								0.02
18–24 years	51.8	5.78	1.00	[Reference]		1.00	[Reference]	
25–49 years	47.7	2.13	0.85	(0.59, 1.22)		0.80	(0.53, 1.19)	
50–64 years	54.2	3.07	1.10	(0.73, 1.66)		1.06	(0.66, 1.70)	
65+ years	53.5	4.44	1.07	(0.66, 1.73)		1.04	(0.60, 1.81)	
Ethnicity					0.01			0.02
White	48.4	1.69	1.00	[Reference]		1.00	[Reference]	
Ethnic minority	63.7	5.15	1.87	(1.29, 2.71)**		1.92	(1.29, 2.85)**	
Clinically relevant indices								
Time since problem resolution					0.00			0.02
0–5 years	48.6	2.37	1.00	[Reference]		1.00	[Reference]	
5–15 years	53.3	3.06	1.21	(0.94, 1.55)		1.16	(0.90, 1.50)	
15+ years	49.6	3.76	1.04	(0.75, 1.46)		0.92	(0.62, 1.35)	
Number of substances ever identified as a problem					0.02			0.03
1 substance	50.6	1.99	1.00	[Reference]		1.00	[Reference]	
2 substances	38.7	3.68	0.62	(0.47, 0.82)**		0.65	(0.49, 0.87)**	
3+ substances	58.7	4.23	1.39	(1.02, 1.88)*		1.47	(1.08, 2.00)*	
Age of onset of first substance					0.00			0.02
<15 years of age	52.3	2.23	1.24	(1.00, 1.53)*		1.32	(1.06, 1.64)*	
≥15 years of age	47.0	2.31	1.00	[Reference]		1.00	[Reference]	
Age of onset of problem substance					0.00			0.02
<15 years of age	51.0	2.59	1.08	(0.87, 1.34)		1.10	(0.89, 1.37)	
≥15 years of age	49.1	2.05	1.00	[Reference]		1.00	[Reference]	
Primary substance					0.04			0.06
Alcohol	46.6	2.06	1.00	[Reference]		1.00	[Reference]	
Cannabis	43.7	3.76	0.89	(0.68, 1.18)		0.94	(0.70, 1.25)	
Cocaine	66.7	6.21	2.30	(1.42, 3.73)**		2.44	(1.50, 3.99)**	
Opioids (heroin, other opiates)	74.2	5.09	3.31	(2.03, 5.39)**		3.21	(1.96, 5.24)**	
Amphetamine	49.0	7.76	1.10	(0.65, 1.88)		1.10	(0.64, 1.89)	
Other (benzodiazepines, hallucinogens, NPS)	59.7	7.17	1.70	(1.06, 2.73)*		1.88	(1.16, 3.05)*	
Lifetime mental health disorder diagnoses								
Alcohol or substance use disorder (versus not)	85.3	2.60	8.86	(6.32, 12.43)**	0.20	9.54	(6.75, 13.49)**	0.21
Mood disorder (versus not)	62.4	3.27	1.95	(1.51, 2.52)**	0.03	2.02	(1.56, 2.62)**	0.04
Anxiety disorder (versus not)	57.5	2.65	1.62	(1.30, 2.03)**	0.02	1.79	(1.42, 2.25)**	0.04
PTSD (versus not)	59.0	4.56	1.52	(1.09, 2.12)*	0.01	1.59	(1.13, 2.22)*	0.04
Other mental health disorder (versus not)	45.4	4.27	0.81	(0.58, 1.13)	0.00	0.86	(0.61, 1.20)	0.02
Never been diagnosed (versus not)	21.9	2.56	0.19	(0.15, 0.25)**	0.14	0.17	(0.13, 0.23)**	0.17
Record of arrest					0.08			0.09
Never been arrested	39.9	2.07	1.00	[Reference]		1.00	[Reference]	
Yes – in the past year	85.3	6.92	8.74	(3.83, 19.98)**		7.88	(3.40, 18.24)**	
Yes – but not in the past year	61.5	2.61	2.40	(1.91, 3.02)**		2.38	(1.88, 3.01)**	

OR, odds ratio; AOR, adjusted odds ratio; NPS, novel psychoactive substance; PTSD, post-traumatic stress disorder.

a. An odds ratio of less than 1 means that participants are less likely to have chosen an assisted pathway.

*P < 0.05, **P < 0.01.

## Discussion

Here we report the first national probability-based estimate of the proportion of UK adults having resolved an AOD problem. The prevalence of 5% equates to 2.7 million people who define themselves as having overcome an alcohol or other drug problem in the UK. As reported in similar work conducted in the USA and Europe,^5,10,11^ we found that AOD problem resolution is not rare and that there are multiple pathways to achieve it. Our survey showed that of those accessing some form of assistance, significant help came from mutual-help groups such as Alcoholics Anonymous or Narcotics Anonymous. Evidence from a treatment population has suggested that rates of 12-step group attendance are lower in UK samples than in the USA,^20^ but these mutual-aid groups are easily accessible and flexible and are free resources that can be found in every region of the UK. Just under one in five participants had seen their primary care physician for their AOD-related problem, and one in four had received specialist AOD treatment (out-patient or in-patient). These services are free at the point of delivery in the UK, although access to the latter has been gradually reduced by financial cuts related to ‘austerity’ from 2010 onwards.^21,22^. Nearly a quarter of respondents reported accessing community-based ‘recovery support services’ that have become more available in the UK since the government Drug Strategy of 2010.^4^ These include ‘recovery housing’ (sober living environments), peer-based recovery support and recovery community centres operated by Lived Experience Recovery Organisations.^19^ This is an emerging form of support that takes a lead from North America and the concept of a recovery-orientated system of care.^23^ Use of licensed medications for the treatment of alcohol and opioid problems was generally low but higher than in the equivalent survey in the USA,^5^ possibly reflecting the difference in healthcare systems between the two countries.

AOD problems occur across a spectrum of use in terms of quantity, frequency and duration.^24^ This study was designed to explore the entire spectrum, framing the screening question in terms of ‘used to have a problem with AOD but no longer do’. AOD problem self-change studies from around the world consistently indicate a better chance of natural recovery among less severe cases.^25–27^ In our study, the strongest correlates of choosing an assisted pathway (as indicated by the model's semi-partial *R*^2^ value, which gives an estimate of the amount of variance explained by that variable on that outcome, independent of other factors in the model) were lifetime diagnosis of an AOD use disorder, having been arrested, and (inversely) never having been diagnosed with any mental health or substance use disorder. There is evidence from the USA that even people with DSM-IV dependence are able to overcome their problem without assistance, with rates of self-change of 25% for alcohol,^13^ 9.7% for heroin^28^ and 56.9% for cannabis.^28^ However, the role of AOD dependence severity is indicated by the greater prevalence of self-change among people with lesser problem severity (i.e. people with less severe problems are more likely to recover on their own). Likewise, the need to overcome a co-existing mental health problem also appears to make self-change more difficult. Certain substances are more likely to require assisted treatment and recovery pathways (cocaine and opiates), whereas others may not require formal treatment, and the individuals might benefit from education and secondary prevention efforts (cannabis).^28^ This may be owing to the physiological impact of the substance, in that medically assisted withdrawal is not required for cannabis, but it may also be linked to the perceived illegality of the substance or other contextual issues. It is important to note that people who had accessed formal addiction treatment may have been more likely to have received a diagnosis than those that did not.

### Strengths and limitations

There are important limitations of this study to consider. The prevalence of recovery depends on the population from which the study sample is drawn. Mellor et al suggest that three different populations exist in the natural recovery literature:^25^ (a) the whole population with a current problem; (b) an untreated population with a problem; and (c) a population that has already achieved remission (i.e. examining the proportion that did so without treatment). Studies such as this one, which produce estimates derived from a sample of individuals that have already achieved remission (i.e. group 3), produce the highest untreated remission rates.^25^ When more stringent definitions of the problem or remission are applied, estimates of untreated remission in remitted samples decrease. Likewise, more inclusive (broader) definitions of treatment decrease estimates of untreated remission derived from remitted samples.^25^ Estimates of untreated remission are higher when treatment is defined as solely formal treatment, as opposed to when the definition includes formal treatment, general treatment and peer-led interventions.^29^

Our screener question left the definition of ‘a drug or alcohol problem’ to the participant, and so no conclusions can be drawn about the proportion of the sample with a diagnosable AOD disorder. This study explores a broader population of individuals who have experienced a variety of self-defined problems with AOD use. It has been well established that a large proportion of individuals who experience health or social consequences of their AOD use do not meet diagnostic criteria for AOD disorder,^30^ and so the findings have importance from a public health perspective. The cross-sectional nature of the design of our study means that caution should be taken regarding any causal connections among variables, and recall biases may have influenced some estimates given the retrospective nature of the data captured. The data and analyses are limited by the lack of detailed information captured about substance use, patterns of treatment use, periods of problem resolution and reoccurrence over time.

### Implications for policy and future research

A two-part independent review of illicit drug use was published in the UK in 2020–2021, providing an up-to-date analysis of the associated problems (part I)^31^ and proposing policy solutions (part II).^21^ The review found that all the main drug-related problems had worsened in the previous decade, including significant increases in the use of opiates and crack cocaine, and in the use of other drugs (particularly cannabis and cocaine) by both adults and children. At the same time, alcohol consumption is the biggest risk factor for death, ill-health and disability among 15–49-year-olds in the UK and the fifth biggest risk factor across all ages.^32^ Against this background of increasing use and worsening harms, the proportion of people who required treatment and actually received it decreased during a decade of financial pressure and service reconfiguration.^21,22^

This study acknowledges the millions of UK residents that report successfully resolving a significant AOD problem and highlights the variety of services and pathways used to do so. Together with AOD problem resolution and recovery estimates from other developed countries,^33^ these data may instil hope and optimism about the chances of recovery for what are traditionally defined as ‘chronically relapsing’ disorders, particularly as more than a third of the sample reported being in stable recovery (i.e. for 5 years or more). If several million people have already successfully overcome problems, it is useful to understand how they did it.^34^

A substantial number of episodes occurred without use of treatment services. Examining how individuals with a history of problematic AOD use successfully resolve such problems without formal help is an important area of investigation from a public health standpoint. A wide variety of barriers to accessing informal^35^ and formal^36^ treatment have been described, assuming that treatment is available in the first place. Klingemann believes that the regular occurrence of self-change, coupled with the general public's lack of awareness of recovery without treatment, suggests that ‘disseminating knowledge about the prevalence of self-change could be a type of intervention itself’.^37^ Demonstrating to the millions of individuals with less severe AOD problems that successful problem resolution is possible without the use of external services may lead to increased self-efficacy and significant population-level change. Many of the strategies used by such individuals are similar to those taught and modelled in formal treatment, such as stimulus control (e.g. avoiding high-risk alcohol/drug-using venues, not keeping alcohol at home), as well as engaging in alternative competing behaviours that are subjectively rewarding, provide structure, and boost agency and self-esteem.^38^ On the other hand, the belief that ‘I should be able to deal with this myself’ is a potential barrier to treatment. It is also worth noting that unassisted recovery does not necessarily mean doing it alone, and support from family, friends and colleagues has a crucial role in building social recovery capital.^39^

In summary, given the somewhat pessimistic and even nihilistic views about successful recovery among the public, policy makers and even clinicians,^40^ understanding, documenting and publicising the reality of addiction recovery in the UK may increase optimism that recovery is possible and also highlight the variety of ways in which this can be achieved.^34^ Once the key pathways to change are identified, this information could be communicated to amplify population-level use of such self-change strategies. Given the dynamic nature of AOD problem resolution, further recovery research will be needed in groups with AOD problems in order to understand who may benefit from what types of services, when, and for what duration and intensity, with a goal of shortening the time to stable remission and recovery. This large population of recovering individuals could be a crucial research resource in discovering the answers to such questions.

## Data Availability

The data that support the findings of this study are available on request from the corresponding author, E.D.
